# Physicochemical and Antioxidant Properties of Gelatin and Gelatin Hydrolysates Obtained from Extrusion-Pretreated Fish (*Oreochromis* sp.) Scales

**DOI:** 10.3390/md19050275

**Published:** 2021-05-14

**Authors:** Wei-Cheng Shiao, Tien-Chiu Wu, Chia-Hung Kuo, Yung-Hsiang Tsai, Mei-Ling Tsai, Yong-Han Hong, Chun-Yung Huang

**Affiliations:** 1 Division of Gastroenterology (General Medicine), Department of Internal Medicine, Yuan’s General Hospital, No.162, Cheng Kung 1st Rd., Lingya District, Kaohsiung 80249, Taiwan; cheng_2034@yahoo.com.tw; 2 Division of Hematology and Oncology, Department of Internal Medicine, Kaohsiung Medical University Hospital, Kaohsiung Medical University, Kaohsiung 80756, Taiwan; 960552@ms.kmuh.org.tw; 3 Department of Seafood Science, National Kaohsiung University of Science and Technology, No. 142, Haijhuan Rd., Nanzih District, Kaohsiung 81157, Taiwan; kuoch@nkust.edu.tw (C.-H.K.); yht@nkust.edu.tw (Y.-H.T.); mltsai@nkust.edu.tw (M.-L.T.); 4 Department of Nutrition, I-Shou University (Yanchao Campus), No.8, Yida Rd., Jiaosu Village, Yanchao District, Kaohsiung 82445, Taiwan

**Keywords:** antioxidant, enzyme digestion, extrusion, fish scales, gelatin, gelatin hydrolysate, *Oreochromis*, pepsin, pancreatin, tilapia

## Abstract

Fish gelatin and its hydrolysates exhibit a variety of biological characteristics, which include antihypertensive and antioxidant properties. In this study, fish gelatins were extracted from extrusion-pretreated tilapia scales, and then subjected to analyses to determine the physicochemical properties and antioxidant activity of the extracted gelatins. Our findings indicate that TSG2 (preconditioned with 1.26% citric acid) possessed the greatest extraction yield, as well as higher antioxidant activities compared with the other extracted gelatins. Hence, TSG2 was subjected to further hydrolyzation using different proteases and ultrafiltration conditions, which yielded four gelatin hydrolysates: TSGH1, TSGH2, TSGH3, and TSGH4. The results showed that TSGH4 (Pepsin + Pancreatin and ultrafiltration < 3000 Da) had a higher yield and greater antioxidant activity in comparison with the other gelatin hydrolysates. As such, TSGH4 was subjected to further fractionation using a Superdex peptide column and two-stage reverse-phase column HPLC chromatography, yielding a subfraction TSGH4-6-2-b, which possessed the highest 2,2-diphenyl-1-picrylhydrazyl (DPPH) scavenging activity compared with the other fractions. Further LC-ESI/MS/MS analysis of TSGH4-6-2-b suggested two novel peptides (GYDEY and EPGKSGEQGAPGEAGAP), which could have potential as naturally-occurring peptides with antioxidant properties. These promising results suggest that these antioxidant peptides could have applications in food products, nutraceuticals, and cosmetics.

## 1. Introduction

Oxidation is a common reaction that occurs in the cells of all living organisms, and therefore, antioxidant activity is an especially important component of normal cellular metabolism [1]. In aerobic organisms, the formation of free radicals is unavoidable. Normal aerobic metabolism involves the production of superoxide anion (^•^O^2^^−^) and hydroxyl radical (^•^OH), which are unwanted metabolic by-products [2]. Free radicals are known to react quickly with other groups and substances, resulting in a wide range of deleterious effects on physiological processes and food [3]. Hence, oxidative stress is thought to play a role in the pathology of a number of human diseases and conditions, including diabetes, atherosclerosis, aging, neurodegenerative disorders, and cancer [4]. As certain synthetic antioxidants, such as butylated hydroxyanisole (BHA), butylated hydroxytoluene (BHT), t-butylhydroquinone (TBHQ), and propyl gallate (PG), may be negatively perceived by consumers and could pose health risks [5], there is a need for antioxidants, especially ones with nutritional and therapeutic properties, that can be derived from naturally-occurring substances [6,7,8,9]. 

Gelatin is widely utilized for a range of applications in the biomedical, pharmaceutical, food, cosmetic, and leather industries [10]. In certain parts of the world, the extraction of gelatin from bovine and porcine by-products is not permitted due to the risk of bovine spongiform encephalopathy, swine foot-and-mouth disease (FMD), or for religious reasons [11]. As a consequence, there has been a gradual increase in the use of fish by-products, including skin, bones, fins, and scales, as alternative sources of gelatin. Fish by-products offer a number of advantages for the production of gelatin, such as proven safety and wide availability. Gelatins containing lower molecular weight peptides such as gelatin hydrolysates have been shown to exhibit greater biological activities [12]. Furthermore, gelatin hydrolysates are valued in the food and pharmaceutical industries due to their ability to benefit various aspects of health, such as reduce oxidation and mitigate hypertension. Thus, there is considerable interest in using gelatin hydrolysates as functional food additives [13]. The use of fish gelatin containing bioactive peptides to produce hydrolysates has been demonstrated to be an effective method of creating value-added materials, and highlights the potential of fish gelatin for other possible applications [14]. 

The process of extrusion involves a high-temperature bioreaction that is short in duration and comprises mixing, heating, shearing, pressurizing, and shaping. During the extrusion process, raw materials are subjected to mechanical shearing at a high temperature and a low moisture content. This results in extruded products with highly modified properties, including microstructure, texture, flavor, and color [15]. It has been shown that extrusion can be used to pretreat rice straw, whereby enzymatic hydrolysis accelerates the saccharification of the rice straw [16]. Soybean dregs can be also extruded to reduce the amount of insoluble dietary fiber (IDF) and increase the soluble dietary fiber (SDF) contained in soybean residues [17]. In our previous study, we pretreated fish scales using extrusion, which enhanced the separation of collagen and hydroxyapatite [8,18]. These results demonstrate that extrusion technology is a powerful tool which can be used to pretreat raw materials, thereby enhancing and shortening the duration of subsequent processing. 

Tilapia is a tropical teleost and one of the most common fish species used in aquaculture in Taiwan. Since 1978, when tilapia farming was first introduced in Taiwan, annual production has steadily increased, and in 2018, tilapia production totaled 65,000 t [19]. In this study, we aimed to explore the potential use of gelatin and gelatin hydrolysates obtained from single-screw extrusion pretreated tilapia scales. The extrusion pretreatment process boosted the extraction yield of fish scale-derived gelatin. In addition, the physicochemical and antioxidant properties of gelatin and its hydrolysates were investigated. To the best of our knowledge, the present study is the first investigation of gelatin hydrolysates obtained from tilapia scales pretreated by single-screw extrusion. Herein, we explore the potential applications of gelatin hydrolysates and discuss the possibility that antioxidant peptides could be of industrial value in the production of food, cosmetics, and nutraceuticals.

## 2. Results and Discussion

### 2.1. Preparation of TSG1-6 from Extrusion-Pretreated Tilapia Scales

Tilapia scales (TS) were obtained from a local fishery located in southern Taiwan. The chemical composition of TS was as follows: 43.8 ± 0.4% protein, 40.0 ± 0.5% ash, 11.5 ± 0.8% water, 4.76 ± 0.8% carbohydrate, and 0.02 ± 0.00% lipid. The ash was predominantly composed of hydroxyapatite [20]. TS contained a large proportion of protein (43.8%), suggesting that it could be a good source of gelatin, a denatured form of collagen. However, collagen contained in fish scales is bound tightly to hydroxyapatite, and thus, it is difficult to separate the two substances. In previous research, we developed an extrusion-pretreatment method that is capable of facilitating the separation of hydroxyapatite and collagen. This process significantly enhances the amount of gelatin that can be extracted from fish scales [18]. The extraction process was performed once the extrudates had been dried. All of the conditions for the extraction of gelatin from TS extrudates were set at 50 °C for 1 h. Table 1 shows that the extruded samples TSG2, TSG4, and TSG6 had significantly greater extraction yields compared with the non-extruded samples (TSG1, TSG3, and TSG5). The fold increases between TSG1 and TSG2, TSG3 and TSG4, and TSG5 and TSG6 were approximately 10.6 (22.2/2.10 = 10.6), 8.85 (19.3/2.18 = 8.85), and 6.10 (13.0/2.13 = 6.10), respectively. Taken together, the data show that TSG2 accounted for the greatest incremental fold increase compared with TSG4 and TSG6. Hence, TSG2 was found to be the best candidate for further development of a commercially produced gelatin owing to its high yield. The extruded samples TSG2, TSG4, and TSG6 exhibited the largest extraction yields, and thus, further analyses were conducted to characterize their physicochemical properties. 

### 2.2. Physicochemical Properties of TSG2, TSG4, and TSG6

The physicochemical characteristics of the gelatin extracts TSG2, TSG4, and TSG6 were analyzed to determine color, viscosity, ash, pH, bloom gel strength, DSC, and fishy odor. The color differences among TSG2, TSG4, and TSG6 were investigated by measuring Hunterlab *L*, *a*, and *b* values, and whiteness index. There were significant differences in *L*, *a*, and *b* values, and whiteness index among the three gelatin extracts (Table 2). TSG6 had the highest whiteness index, followed by TSG2 and TSG4. These findings show that pretreatment by NaOH and preconditioning by citric acid induced an observable whitening of the gelatin extracts. Whiter gelatin extracts generally have better commercial potential. Regarding ash contents, the values for TSG2, TSG4, and TSG6 were 1.75% ± 0.06%, 3.58% ± 0.27%, and 3.33% ± 0.09%, respectively (Table 2). Overall, TSG2 exhibited the lowest ash content, indicating it had the greatest purity among the extracted gelatins, and the pH values (in 6.67% solution) of TSG2, TSG4, and TSG6 were 7.2, 6.2, and 9.9, respectively (Table 2). The highest pH value seen in TSG6 could be attributable to the 0.1 N NaOH pretreatment. The quality of a gelatin is primarily assessed according to its gel strength [18]. Differences in gel strength among gelatin extracts can potentially be attributed to fish species, biomaterial (skin, bone, or scales), composition of amino acids, and the size of protein chains [21]. In this study, TSG6 exhibited the greatest bloom gel strength (202 ± 0 g), followed by TSG2 (185 ± 5 g) and TSG4 (157 ± 5 g) (Table 2). Compared with previous research, the gel strength of TSG2 and TSG6 in the current research was superior to the most commonly reported fish-derived gelatins, including Nile perch bone (179 and 134 g, respectively, for young and adult fish) [21], Atlantic salmon skin (108 g) and cod skin (71 g) [22], giant catfish skin (153.0 g) and calf skin (134.8 g) [23], bigeye snapper skin (105.7 g) [24], and sin croaker (125 g) and shortfin scad (177 g) [25]. As such, the high gel strength of TSG2 and TSG6 suggests they may have potential for commercial development. Table 2 displays the viscosity of gelatin sample solutions TSG2, TSG4, and TSG6. TSG2 and TSG6 exhibited higher viscosity compared with TSG4, indicating that TS preconditioning using citric acid solution can enhance its viscosity. The viscosity of gelatin samples may vary according to molecular weight and molecular size distribution of proteins [26]. Viscosity is regarded as the second most important commercial physical characteristic of collagen-based products [27]. Typically, gelatins with a higher viscosity have a greater potential for commercial use. The viscosities of the extracted gelatins TSG2, TSG4, and TSG6 ranged from 8.10 to 9.77 cps (measured at a concentration of 6.67% and a temperature of 60 °C), as compared to previously reported results from lizardfish scales, which ranged from 3.14 to 5.80 cps (concentration 6.67%; temp 25 °C) [28]. Although the measuring temperatures were different, gelatin solution at higher temperatures normally possesses lower viscosity, and thus, TSG2, TSG4, and TSG6 had greater viscosities than those extracted from lizardfish scales. The DSC results of TSG2, TSG4, and TSG6 are shown in Table 2. DSC analysis can be performed using rehydrated samples (rehydrated in 0.05 M acetic acid at a sample/solution ratio of 1:40 (*w*/*v*)) [29] or solid state [30]. When a sample is analyzed in a solid state, there are usually two endothermal peaks (*T*_max_ = 77 and 121 °C) [30]. In the present investigation, we utilized the solid state of gelatins for DSC analysis. It was found that two distinct endothermal peaks on the DSC thermograms of TSG2, TSG4, and TSG6 (Table 2). The first peak (*T*_max1_) can be attributed to the thermal denaturation temperature of the sample, while the second peak (*T*_max2_) is attributable to the ongoing conformational changes and subsequent destruction of materials [30]. The comparatively high denaturation parameters of *T*_max1_ and H1 may explain the high thermal stability of collagenous products. In Table 2, the *T*_max1_ of TSG2 (79.0 °C), TSG4 (57.9 °C), and TSG6 (62.6 °C) are displayed. The total H1 values of TSG2, TSG4, and TSG6 were 1.40, 8.01, and 3.37 J/g, respectively. A greater *T*_max1_ was observed in TSG2 compared with that found in TSG4 and TSG6. The higher thermal stability of TSG2 indicates that it would have a broader range of potential applications. Moreover, the *T*_max2_ values among TSG2, TSG4, and TSG6 were not markedly different. The odor and overall acceptability of gelatin extracts TSG2, TSG4, and TSG6 were assessed using a nine-point hedonic scale, with 1 indicating “very bad” and 9 indicating “very good.” The results shown in Table 2 demonstrate that for odor (in solution), odor (in powder), and overall acceptability, gelatin samples TSG2 (preconditioned with citric acid solution) and TSG6 (preconditioned with citric acid solution) had better hedonic scores compared with TSG4 (preconditioned with acetic acid solution). These results clearly show that TS preconditioned using citric acid solution exhibited superior odor acceptability compared with that achieved with acetic acid solution. We speculate that, due to the distinctive pungent odor of acetic acid, the TSG4 adversely enhanced the unpleasant odor, and thus, received the lowest overall odor acceptability. In summary, TSG2 was found to have high values for whiteness, gelatin purity, bloom gel strength, viscosity, thermal stability, and odor acceptability. Hence, TSG2 would be the obvious choice for further commercial development.

### 2.3. Structure and Molecular Weights of Extracted Gelatins TSG2, TSG4, and TSG6

**Figure 1:**
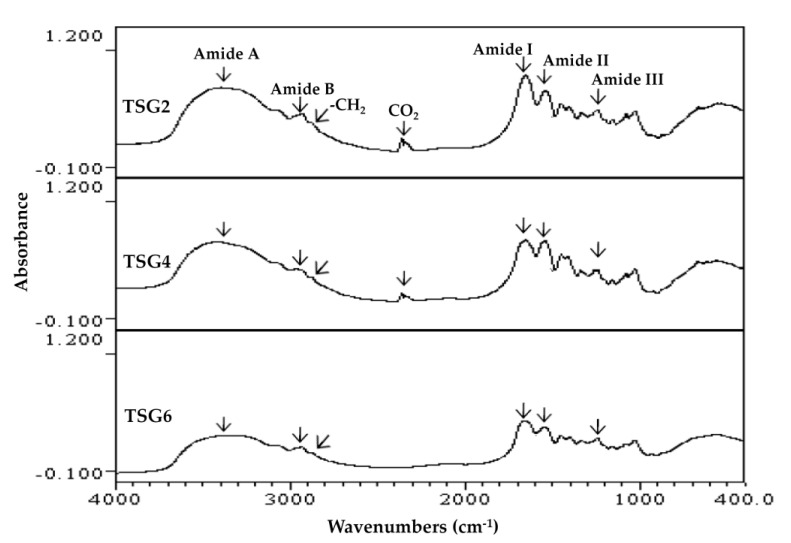
FTIR spectra of TSG2, TSG4, and TSG6. Absorption bands at amide A, amide B, -CH_2_, CO_2_, amide I, amide II, and amide III are indicated.

**Figure 2:**
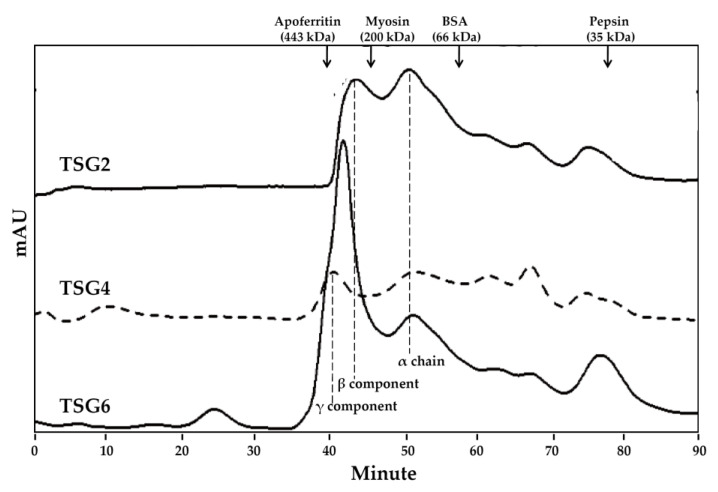
Size exclusion chromatographic profiles of TSG2, TSG4, and TSG6. Apoferritin (443 kDa), myosin (200 kDa), BSA (66 kDa), and pepsin (35 kDa) were used as standard proteins.

FTIR and HPLC gel filtration chromatography were used to analyze the structure and MW profiles of TSG2, TSG4, and TSG6. It has been shown previously that the FTIR spectra of collagenous products exhibit five major absorption bands in the amide band region, as follows: 3304–3315 cm^−1^ (amide A: NH stretch, coupled with hydrogen bonding), 2922–2940 cm^−1^ (amide B: asymmetric stretching vibration of =C-H and –NH_3_^+^), 1644–1653 cm^−1^ (amide I: stretching vibration of C=O involved in hydrogen bonds), 1541–1548 cm^−1^ (amide II: NH bend, coupled with CN stretch), and 1237–1239 cm^−1^ (amide III: NH bend) [31,32,33,34]. The amide A, amide B, and -CH_2_ stretch (2853 cm^−1^) bands showed a tendency to join (Figure 1), which may be due to the dimeric intermolecular associations of carboxylic groups [33]. In the present study, the FTIR spectra of TSG2, TSG4, and TSG6 showed the distinct characteristic peaks of amides A and B, as well as amides I, II, and III (Figure 1). There was little difference in the major absorption bands at amide A, amide B, amide I, amide II, and amide III among TSG2, TSG4, and TSG6, suggesting that their structural properties were not notably changed. In addition, the band at 2355 cm^−1^ was visible in TSG2 and TSG4, which may correspond to carbon dioxide adsorption on the sample surface [35]. The MW profiles of TSG2, TSG4, and TSG6 are displayed in Figure 2. The α chain (approximately 116.5 kDa) and β component (approximately 255.5 kDa) were observed in TSG2, the α chain (approximately 116.5 kDa) and γ component (approximately 417.3 kDa) were visible in TSG4, and the α chain (approximately 116.5 kDa) and γ component (approximately 417.3 kDa) were detected in TSG6. Taken together, the results of our analyses of TSG2, TSG4, and TSG6 and the characteristic MW profiles of these gelatin samples were consistent with previously reported findings [18]. 

### 2.4. Antioxidant Activities of TSG2, TSG4, and TSG6

**Figure 3:**
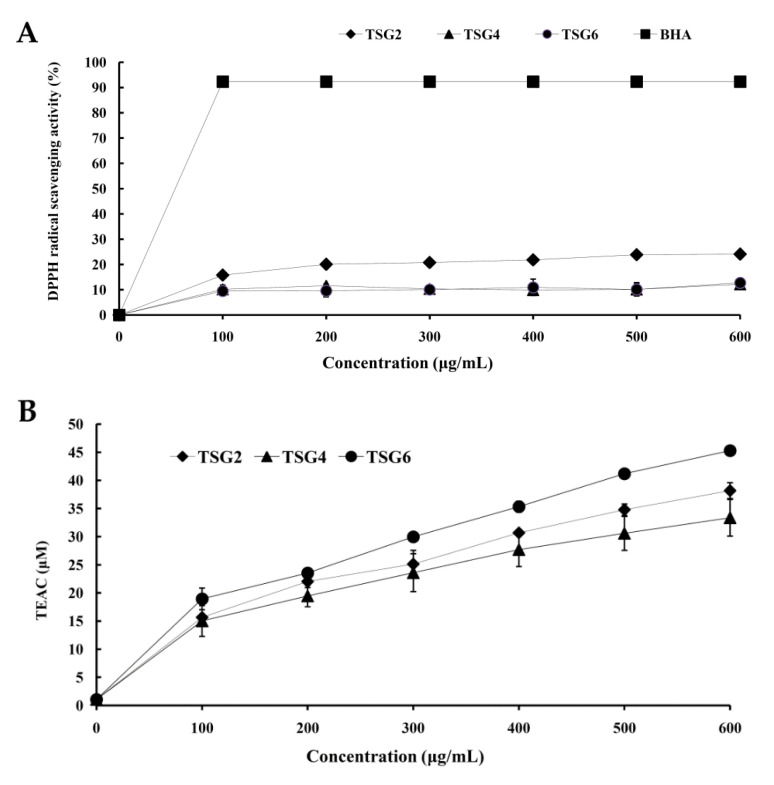

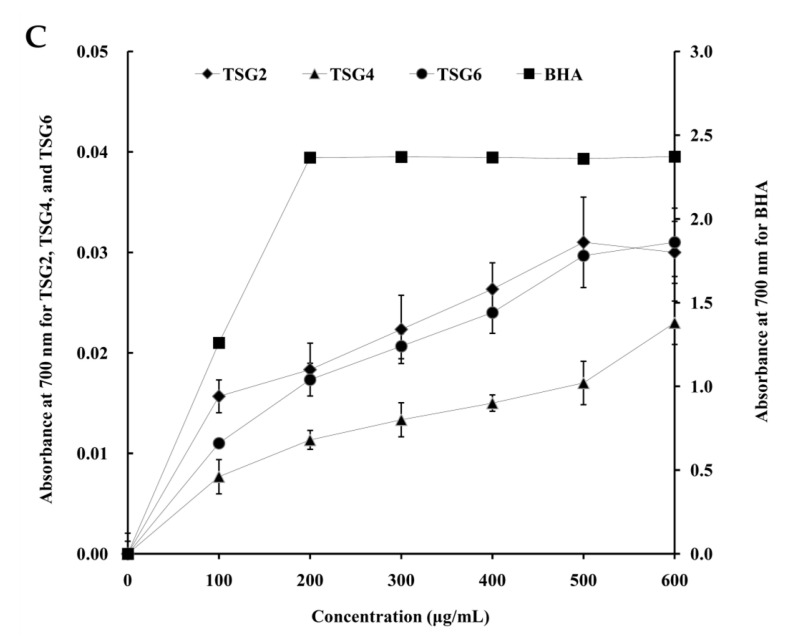
Antioxidant activities of TSG2, TSG4, and TSG6. (**A**) DPPH radical scavenging activities of TSG2, TSG4, TSG6, and BHA. (**B**) ABTS radical scavenging activities of TSG2, TSG4, and TSG6. (**C**) Reducing power of TSG2, TSG4, TSG6, and BHA.

The antioxidant properties of TSG2, TSG4, and TSG6 were assessed using 2,2-diphenyl-1-picrylhydrazyl (DPPH), 2,20-azino-bis (3-ethylbenzothiazoline-6-sulphonic acid) diammonium salt (ABTS), and reducing power analyses. Figure 3A shows the scavenging effects of TSG2, TSG4, and TSG6 and BHA (positive control) on DPPH free radicals. DPPH scavenging characteristics were detected in all of the gelatin extracts, TSG2, TSG4, and TSG6. Moreover, TSG2 exhibited greater DPPH radical scavenging activity compared with those of TSG4 and TSG6. ABTS radical assay is a powerful analytic tool that is capable of measuring levels of hydrogen-donating antioxidants. The technique employs a process whereby the radical is quenched forming an ABTS radical complex [36,37]. Figure 3B shows the ABTS^∙+^ scavenging activities of gelatin extracts TSG2, TSG4, and TSG6. All of the gelatin extracts TSG2, TSG4, and TSG6 exhibited dose-dependent ABTS^∙+^ scavenging activities. Overall, TSG6 showed the greatest ABTS^∙+^ scavenging activity, followed by TSG2, with TSG4 having the least activity. The reducing power of a substance is largely related to the presence of reductones (antioxidants), which donate hydrogen atoms, resulting in disruption of the free radical chain, and this process confers an antioxidant effect. Antioxidant components in samples lead to a reduction of the Fe^3+^/ferricyanide complex to the Fe^2+^ form, which can be assessed by detecting the formation of Prussian blue at 700 nm [38]. The reducing power of gelatin extracts TSG2, TSG4, and TSG6 and BHA (positive control) is shown in Figure 3C. Great reducing power was found in all of the gelatin extracts and increased in proportion to their concentration. TSG2 had the greatest reducing power, followed by TSG6 and TSG4, despite the lack of any significant difference (*p* > 0.05) between TSG2 and TSG6. In summary, gelatin extracts that were preconditioned using citric acid (TSG2 and TSG6) had higher antioxidant activities compared with those prepared using acetic acid (TSG4). Among the tested gelatin extracts, TSG2 exhibited the strongest antioxidant activities and was, therefore, selected for further analyses. 

### 2.5. Preparation of Gelatin Hydrolysates TSGH1, TSGH2, TSGH3, and TSGH4 and Analyses of Antioxidant Activity 

Enzymatic hydrolysis is a commonly used tool in the production of food-grade protein hydrolysates. This process results in the release of bioactive peptides from their protein precursors [8]. It is an environmentally friendly technique because it requires little to no toxic chemicals or solvents. In the current study, the gelatin extract TSG2 was selected for hydrolysis using pepsin and pancreatin (industrial food-grade proteinases) in order to obtain gelatin hydrolysates. Table 3 shows the results of the four gelatin hydrolysates, (TSGH1, TSGH2, TSGH3, and TSGH4) obtained using different enzymes and ultrafiltration conditions. The size of MW is thought to be the most important determinant of the biological functions of protein hydrolysates [39]. It can be seen in Table 3 that for TSGH1, the major MW distributions ranged from 2883–37,466 Da and the peak MW was 24,012 Da; for TSGH2 the range was 1800–37,466 Da, with a peak MW of 18,006 Da; TSGH3 ranged from 96–1184 Da and the peak MW was 131 Da; for TSGH4, range was 74–21,625 Da, with a peak MW of 1315 Da. The aforementioned results demonstrate that the MWs of TSGH1 (ultrafiltration > 3000 Da) and TSGH2 (ultrafiltration > 3000 Da) were greater and the peak MWs were located at 24,012 and 18,006 Da, respectively. However, TSGH3 (ultrafiltration < 3000 Da) and TSGH4 (ultrafiltration < 3000 Da) had smaller MWs with peaks located at 131 and 1315 Da, respectively. Furthermore, the gel filtration graph (Supplementary Figure S1) of TSGH1-4 shows the TSGH3 yield was very low, indicating that for hydrolysis of TSG2, pepsin alone is insufficient. In contrast, TSGH4 was obtained from the digestion of TSG2 by two enzymes (pepsin + pancreatin), and thus, it had a higher production yield. In addition, DPPH, ABTS, and reducing power assays were used to assess the antioxidant activities of TSGH1, TSGH2, TSGH3, and TSGH4. Table 3 shows that, in general, the samples with larger amounts of low-molecular weight hydrolysates, i.e., TSGH3 and TSGH4, possessed greater DPPH and ABTS scavenging activities and reducing power compared with the other samples, i.e., TSGH1 and TSGH2. Compared with TSGH3, TSGH4 had a greater production yield and was, thus, selected for further HPLC fractionation experiments. 

### 2.6. Preparation of Fractionated Products using TSGH4 and Assessment of Their DPPH Scavenging Activities 

**Figure 4:**
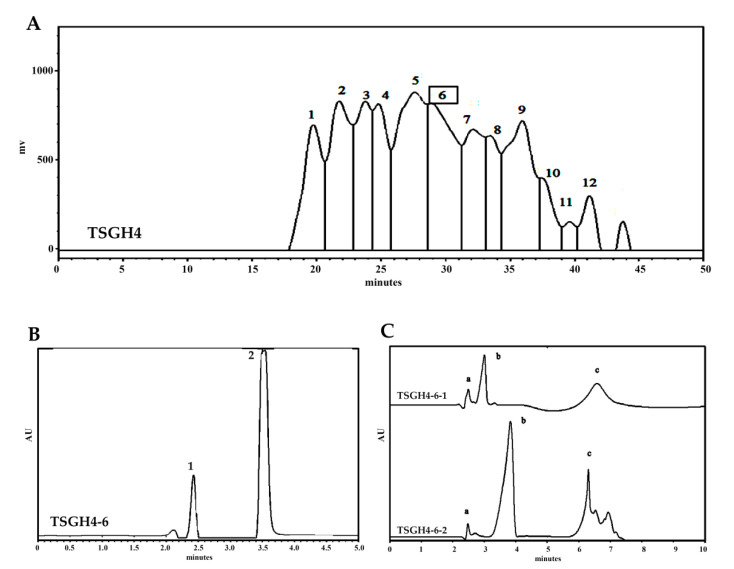
HPLC profiles for gelatin hydrolysates. (**A**) Superdex peptide chromatographic profiles of TSGH4. (**B**) RP-HPLC chromatographic profiles of TSGH4-6. (**C**) RP-HPLC chromatographic profiles of TSGH4-6-1 and TSGH4-6-2.

Gel filtration chromatography was used to separate the fractions with different MW distributions from TSGH4. In total, 12 fractions (TSGH4-1–TSGH4-12) were collected (Figure 4A) using gel filtration chromatography. These fractions were then assayed to determine their DPPH scavenging activity (Table 4). In the present study, we employed the DPPH free radical scavenging method to assess the antioxidant activities of fractionated gelatin hydrolysates due to the greater stability of free radicals and a short assay time [40]. The results showed that TSGH4-6 had the greatest DPPH scavenging activity among the 12 fractions that were analyzed (Table 4). RP-HPLC is an effective technique that uses the hydrophobic/hydrophilic peptide ratio to separate peptides in protein hydrolysates [41]. Two fractions (TSGH4-6-1 and TSGH4-6-2) were collected (Figure 4B) following RP-HPLC chromatography and were assayed to determine their DPPH scavenging activity (Table 4). The results showed DPPH scavenging activities in TSGH4-6-1 and TSGH4-6-2 were similar (Table 4). Hence, a second-run RP-HPLC chromatographic analysis of TSGH4-6-1 and TSGH4-6-2 was conducted. Three individual fractions (TSGH4-6-1-a, TSGH4-6-1-b, and TSGH4-6-1-c for TSGH4-6-1; and TSGH4-6-2-a, TSGH4-6-2-b, and TSGH4-6-2-c for TSGH4-6-2) were collected (Figure 4C) after RP-HPLC chromatography, and DPPH scavenging activity was assayed (Table 4). The results in Table 4 show that TSGH4-6-2-b had the greatest DPPH scavenging activity among the tested fractions, despite not being significantly different (*p* > 0.05) compared with those of TSGH4-6-1-b and TSGH4-6-2-c. In summary, following fractionation, the fractionated gelatin hydrolysates showed enhanced DPPH scavenging activities. TSGH4-6-2-b, which had the most potent DPPH scavenging activity, was selected for further LC-ESI/MS/MS analysis. 

### 2.7. Potential Antioxidant Peptides in TSGH4-6-2-b by LC-ESI/MS/MS Analysis

The fractionated gelatin hydrolysate TSGH4-6-2-b exhibited the greatest DPPH scavenging activity and was, thus, subjected to further LC-ESI/MS/MS analysis. The results are displayed in Table 5. The most intense ions in the hydrolysate exhibited homology with the collagen molecules (collagen alpha-2(I) chain and collagen type I alpha 1). Moreover, Gly and Pro were the two major amino acids detected in the peptide sequences. Peptides responsible for the scavenging activity serve as electron donors and convert free radicals to more stable products and terminate the radical chain reaction [42]. The presence of certain amino acids in the hydrolysates increase the scavenging activities of peptides. Among the peptides, Trp, Tyr, and Met had the strongest antioxidant activity, followed by Cys, His, and Phe [43]. Furthermore, it was previously demonstrated that among the antioxidative peptides obtained from the enzymatic hydrolysates of tuna dark muscle by-product, Tyr, Pro, Glu and Asp were the key variables correlated with antioxidant activity [44]. Gelatin subfractions with peptides containing large amounts of Arg, Tyr, and Phe are also thought to possess higher antioxidant activity [12]. In addition, the presence of acidic amino Glu in the peptides could induce strong antioxidant effects, as a free electron is available for interaction with free radicals [45]. Peptides composed of hydrophobic amino acids coupled with Pro, Gln, Glu, and Ser have a strong antioxidant activity in the peptide sequence [46]. Based on these results, we suggested two peptide sequences (GYDEY and EPGKSGEQGAPGEAGAP) (Table 5) containing more of the abovementioned antioxidative amino acids, which could have potential as novel antioxidant peptides. Further research on the synthesis of the two novel antioxidant peptides and conducting in vivo experiments are required for confirmation of these findings. 

## 3. Materials and Methods

### 3.1. Materials and Chemicals

Tilapia fish scales were obtained from a fishery in southern Taiwan. Fresh fish scales were kept on ice and then immediately transported to our laboratory. Some of the fish scales were mixed with 0.1 N NaOH, and the mixture was then washed with tap water to attain a pH of 7.0. All of the fish scales were dried at 50 °C until a moisture content of less than 10% was obtained. Dried fish scales were then milled into a powder (<20 mesh) and stored at room temperature in aluminum foil bags until use. Pepsin, apoferritin, pancreatin, trypsin, myosin, aprotinin, glycine, glutathione, Gly-Gly-Gly, potassium ferricyanide, potassium bromide (KBr), trichloroacetic acid, DPPH, ABTS, 6-hydroxy-2,5,7,8-tetramethylchroman-2-carboxylic acid (Trolox), and bovine serum albumin (BSA) were purchased from Sigma-Aldrich (St. Louis, MO, USA). Superdex 200 10/30 column (300 mm × 10 mm ID) and Superdex Peptide HR 10/30 column (300 mm × 10 mm ID) were purchased from GE Healthcare (Piscataway, NJ, USA). Inspire C18 column (4.6 mm × 250 mm, 5 μm) was purchased from Dikma Technologies Inc. (Lake Forest, CA, USA). All of the other chemicals used in experiments were of analytical grade and were purchased from Sigma-Aldrich (St. Louis, MO, USA). 

### 3.2. Extrusion-Cooking Pretreatment

The extrusion operation variables are shown in Table 1. For TSG5 and TSG6, the fish scales were pretreated with 0.1 N NaOH prior to extrusion, which has been demonstrated to remove non-collagenous proteins and eliminate any undesirable fishy odors [47]. Following pretreatment with 0.1 N NaOH, TSG5 and TSG6 were washed with water until the pH was neutral or slightly basic. Fish scale powder was utilized as the raw material and preconditioned by mixing with 1.26% citric acid or 9.37% acetic acid such that the final moisture content was 27%. Extrusion cooking was conducted using a model single-screw extruder (Tsung Hsing Food Machinery, Kaohsiung, Taiwan) that was equipped with a screw diameter of 74 mm, a screw length-to-diameter (L/D) ratio of 3.07:1, and a rounded die opening (3 mm) at the end of the extruder. Heating of the barrel was controlled using an electric heating element which jacketed the barrel and thermal probe. The barrel temperature was set at 135 °C. The screw speed was kept constant at 360 rpm. The feed rate was set at 11.4 kg/h to ensure stable operation of the extruder. The extrudate was collected at the die end and kept at 50 °C in a hot air oven for 30 min in order to remove excess moisture. Once the extrusion-cooking procedure had been completed, the extrudate was ground into fine particles (<20 mesh) and sealed in aluminum foil bags. It was stored at 4 °C until further use. 

### 3.3. Extraction of Gelatin from Non-Extruded Fish Scales and Fish Scale Extrudate

The extraction operation variables are shown in Table 1. Non-extruded fish scale powder or fish scale extrudate powder was soaked in ddH_2_O using a sample ratio of 1:10 (w/v) and was then shaken in a water bath at 50 °C for 1 h. The mixture was centrifuged at 10,200 × *g* for 10 min. The supernatant was collected and subjected to determination of its protein concentration using the Lowry method. It was then lyophilized and the gelatin products were obtained. The gelatin yield was expressed as the weight of the protein extracted by hot water/weight of crude protein fish scale content (dry basis) using Equation (1):

Yield (%) = [(protein content of supernatant (g/mL) × volume of supernatant (mL))/(weight of crude protein content of fish scales (g), dry basis)] × 100(1)

### 3.4. Determination of Protein Concentration

The Lowry assay was performed as described previously [48]. Calibration was conducted using different concentrations of stock BSA protein solution (1 mg/mL).

### 3.5. Color Analysis

The color of the gelatin samples that had been generated using various operating conditions were determined. Tristimulus color values, i.e., *L* (lightness), *a* (redness-greenness), and *b* (yellowness-blueness) values, were assessed using an SA-2000 spectrophotometer (Nippon Denshoku, Tokyo, Japan). The data were recorded using at least three separate gelatin samples for each test point. The whiteness index (WI) was calculated using Equation (2):

Whiteness index (WI) = 100 − ((100 − *L*)^2^ + *a*^2^ + *b*^2^)^0.5^ (2)

### 3.6. Chemical Composition Analyses

The measurements of the moisture, fat, ash, and crude protein content were carried out using the following AOAC (1984) procedures [49]: moisture (%) was measured by drying samples in an oven at 103 °C for 8 h; crude fat (%) was determined gravimetrically after extraction of Soxhlet with petroleum ether; crude ash (%) was obtained by incineration in a muffle furnace at 580 °C for 8 h; and crude protein (N × 5.95) (%) was assessed using the Kjeldahl method following acid digestion. 

### 3.7. Determination of Gel Strength

The bloom gel strength was analyzed using the British Standard 757: 1975 method [50] with a texture analyzer (Model TA1000, Stevens LFRA, Harlow, UK). A solution containing 6.67% (*w*/*v*) gelatin sample was prepared by mixing 7.5 g of gelatin and 105 mL of ddH_2_O in a Bloom bottle. The mixture was stirred and allowed to stand for 30 min at room temperature to allow the gelatin to absorb water and swell. The Bloom bottles were transferred to a water bath and maintained at 42 °C for 30 min with intermittent stirring. The samples were then transferred to a cold water bath, which was maintained at 10 ± 0.1 °C. The samples were kept at this temperature for 16–18 h before evaluating the gel strength. The Bloom gel strength (in g) was assessed with the texture analyzer set to generate a 4 mm depression at a rate of 0.5 mm/s.

### 3.8. Differential Scanning Calorimetry (DSC)

A sample of lyophilized protein weighing 5 mg was accurately weighed and placed on an aluminum pan, hermetically sealed, and then scanned from 0 to 200 °C at a heating rate of 5 °C/min. An empty sealed aluminum pan served as a reference. Software was used to record the maximum denaturation temperature (*T*_max_), which was deemed to be the peak temperature of each endothermic peak. The total denaturation enthalpy (ΔH) (J/g protein sample) was assessed for each peak by calculating the corresponding area under each of the endothermic peaks. The analysis was conducted using a DSC 200 F3 calorimeter (Netzsch-Gerätebau GmbH, Selb, Germany), which was calibrated for temperature and enthalpy with indium as the standard. The measurements were performed while samples were being continuously purged using ultra-high-purity nitrogen at 50 cm^3^/min [18].

### 3.9. Determination of Viscosity

The analysis was based on a previous report [51] with some modification. In brief, each of the gelatin samples (6.67%, *w*/*v*) was placed in a water bath maintained at 45 °C until it had melted. The viscosity was recorded at 60 °C using a DV-II + PRO viscometer (Brookfield, MA, USA). The measurements were carried out in triplicate. 

### 3.10. Sensory Evaluation

The sensory evaluation was performed using a 24-member panel. Individuals capable of detecting off-odor in samples with a slightly putrid odor were selected for this evaluation. Samples used for the sensory evaluation were prepared using lyophilized gelatin powder. The samples (in solution 6.67%; *w*/*v* or in powder) were placed in test tubes with screw caps. The samples in solution were kept in a water bath at 50 °C with the screw caps lightly tightened. In each assessment, panelists were instructed to remove the screw caps and then smell the contents. They were then required to identify the odor that they perceived and record the odor acceptability. A nine-point hedonic scale (1 = very bad; 5 = moderate; 9 = very good) was employed for the evaluation of odor acceptability. 

### 3.11. Fourier Transform Infrared (FTIR) Spectroscopy

A volume of protein powder measuring 2 mg was evenly ground with approximately 100 mg KBr until the particles measured less than 2.5 μm in size. The transparent KBr pieces were generated at 500 kg/cm^2^ under vacuum condition. An FT-730 spectrometer (Horiba, Kyoto, Japan) was used to obtain the FTIR spectra. The signals were collected automatically using 60 scans covering the range 4000–400 cm^−1^ at a resolution of 2 cm^−1^ and were compared to a background spectrum collected using KBr alone.

### 3.12. Preparation of Gelatin Hydrolysates

Two proteases, pepsin and pancreatin, were used for the digestion of gelatin. For the digestion of gelatin using pepsin, gelatin (4.5 g) was dissolved in a 150 mL 0.1 M KCl-HCl buffer solution (pH = 2.0), and then hydrolyzed with pepsin (protein to pepsin ratio was 25:1 (*w*/*w*)) for 4 h at 37 °C in a batch reactor. The pH was adjusted to 7.0 by 2 N NaOH, and then the resulting solution was heated at 98 °C for 10 min in order to inactivate the protease. For the digestion of gelatin using pepsin + pancreatin, the gelatin (4.5 g) was dissolved in a 150 mL 0.1 M KCl-HCl buffer solution (pH = 2.0), and hydrolyzed with pepsin (the protein to pepsin ratio was 25:1 (*w*/*w*)) for 4 h at 37 °C in a batch reactor. The pH was adjusted to 7.0 by 2 N NOH, and further hydrolyzed using pancreatin (the protein to pancreatin ratio was 25:1 (*w*/*w*)) for 4 h at 37 °C in a batch reactor. The resulting solution was heated at 98 °C for 10 min in order to inactivate the protease. The abovementioned gelatin hydrolysates were cooled using cold flowing water, and then separated using an ultrafiltration membrane with a molecular weight cut-off (MWCO) of 3000 Da. Both the retentate and permeate fractions were subsequently collected and lyophilized. A summary of the operational variables for enzymatic digestion of TSG2, and four gelatin hydrolysates, namely TSGH1 (digested by pepsin and MWCO > 3000 Da), TSGH2 (digested by pepsin + pancreatin and MWCO > 3000 Da), TSGH3 (digested by pepsin and MWCO < 3000 Da), and TSGH4 (digested by pepsin + pancreatin and MWCO < 3000 Da) can be viewed in Table 3. 

### 3.13. Molecular Weight Analysis

With respect to the molecular analysis of the gelatin samples, a size exclusion HPLC column Superdex 200 (300 mm × 10 mm ID) (GE Healthcare Life Sciences, Chicago, IL, USA) was used, which utilized a Shimadzu HPLC system (Shimadzu, Kyoto, Japan). The conditions of chromatography were as follows: eluent 0.02 M sodium phosphate and 0.25 M sodium chloride, at pH 7.2; flow rate 0.4 mL/min, sample concentration 1%; injection volume 100 μL; temperature 25 °C; and wavelength 280 nm. The standard proteins used to calibrate MW were apoferritin (443 kDa), myosin (200 kDa), BSA (66 kDa), and pepsin (35 kDa). The molecular analysis of gelatin hydrolysate was performed using a size exclusion HPLC column Superdex Peptide HR 10/30 column (300 mm × 10 mm ID) (GE Healthcare Life Sciences, Chicago, IL, USA) with a Shimadzu HPLC system (Shimadzu, Kyoto, Japan). The conditions of chromatography were as follows: eluent 0.02 M sodium phosphate and 0.25 M sodium chloride, at pH 7.2; flow rate 0.5 mL/min, sample concentration 1%; injection volume 50 μL; temperature 25 °C; and wavelength 214 nm. The standard proteins used for calibration of MW were BSA (66 kDa), trypsin inhibitor (20 kDa), aprotinin (6511 Da), glutathione (307 Da), gly-gly-gly (189 Da), and glycine (75 Da). 

### 3.14. Two-Step Separation of Gelatin Hydrolysates by Reversed-Phase High-Performance Liquid Chromatography (RP-HPLC)

A Shimadzu HPLC system (Shimadzu, Kyoto, Japan) equipped with an Inspire C18 column (250 × 4.6 mm, 5 μm) was utilized to separate gelatin hydrolysates. In the first-step of the separation process, the mobile phase was 30% acetonitrile (ACN)/0.1% trifluoroacetic acid (TFA). A volume of gelatin hydrolysates measuring 50 μL was injected into the column and eluted with the mobile phase at a flow rate of 1 mL/min. The separation was monitored at a wavelength of 214 nm and the fractions were collected every minute and used for additional experiments. In the second-step separation, solvent A was 0.05% TFA and solvent B was 100% ACN. A volume of peptides measuring 100 μL was eluted using a linear gradient from 0% to 50% of solvent B for 5 min at a flow rate of 1 mL/min. The separation was monitored at a wavelength of 214 nm and the fractions were collected every minute for additional experiments. 

### 3.15. DPPH Radical Scavenging Activity

The DPPH radical scavenging activity was assessed using a protocol described elsewhere [52]. A volume measuring 50 μL was added to 200 μL 0.1 mM DPPH solution (in methanol). The mixture was then vortexed for 1 min and left in the dark for 30 min at room temperature. Next, the absorbance of the solutions was measured at 517 nm using a PowerWave 340 ELISA reader (Bio-Tek Instruments, Winooski, VT, USA). Equation (3) was used to assess the scavenging activity of the DPPH radicals:

Scavenging activity (%) = (1 − A_sample_/A_control_) × 100 (3)

where A_control_ represents the absorbance of the methanol solution of DPPH without the sample and A_sample_ denotes the absorbance of the methanol solution of DPPH with the tested samples. 

### 3.16. ABTS Radical Scavenging Activity

The ABTS radical scavenging activity was evaluated according to method described elsewhere [53]. The ABTS reagent was generated by mixing 5 mL of 7 mM ABTS solution with 88 μL of 140 mM potassium persulfate in the dark at room temperature for 16 h in order to complete the formation of radicals. The solution was then diluted with 95% ethanol such that the absorbance at 734 nm fell within the range 0.70 ± 0.05. To analyze the scavenging activity, 100 μL ABTS reagent was mixed with 100 μL of each sample solution. The mixture was reacted at room temperature for 6 min and the absorbance was read at 734 nm using a PowerWave 340 ELISA reader (Bio-Tek Instruments). The blank was prepared according to the same protocol using distilled water rather than the sample. Equation (4) was used to calculate the scavenging activity of ABTS radicals:

Scavenging activity (%) = (1 − A_sample_/A_control_) × 100 (4)

where A_control_ denotes the absorbance of ABTS without the sample and A_sample_ represents the absorbance of ABTS with the tested samples. Trolox was prepared as a standard. A calibration curve showing the scavenging percentage against the various concentrations of Trolox standard was created. The ABTS radical scavenging activity of the samples was expressed as Trolox equivalent antioxidant capacity (TEAC), indicating the concentration (μM) of Trolox.

### 3.17. Reducing Power Assay

The reducing power was evaluated according to a previously described protocol [54]. In brief, 0.5 mL of the sample was mixed with 0.5 mL of phosphate buffer (0.2 M, pH 6.6) and 0.5 mL of potassium ferricyanide (1%). The mixture was then incubated at 50 °C for 20 min, followed by addition of 0.5 mL of trichloroacetic acid (10%) and centrifugation (970× *g* for 10 min). Finally, 0.5 mL of the supernatant solution was mixed with 0.5 mL of ddH_2_O and 0.1 mL of FeCl_3_ (0.1%), and allowed to stand for 10 min. The absorbance was measured at 700 nm using a PowerWave 340 ELISA reader (Bio-Tek Instruments). Increased absorbance of the reaction mixture indicated increased reducing power. 

### 3.18. LC-ESI/MS/MS and Data Analyses

An UltiMate 3000 RSLCnano LC Systems (Thermo Fisher Scientific, San Jose, CA, USA) connected to a TripleTOF^®^ 6600 System (Applied Biosystems Sciex, Framingham, MA, USA) equipped with a nanoelectrospray ion source was used. The peptides, which had been dissolved in ddH_2_O, were desalted in a C18 trap column (100 μm × 2 cm nanoViper, 3 μm, 100 Å, Thermo Fisher Scientific) for 4.5 min at a flow rate of 10 μL/min. Samples were subsequently dried and redissolved in ddH_2_O containing 0.1% FA. Aliquots (10 μL) were then injected using an autosampler. Next, the sample was separated using a C18 resolving column (75 μm I.D. × 25 cm nanoViper, 2 μm, 100 Å, Thermo Fisher Scientific) at a flow rate of 300 nL/min. The mobile phases were composed of water with 0.1% formic acid (A) and 100% acetonitrile with 0.1% formic acid (B), respectively. The peptides were separated using a linear gradient of 5% to 30% B over 90 min, followed by 30% to 60% for 6 min, and 60% to 90% for an additional 6 min. The mass spectrometer readings were done using the information-dependent acquisition (IDA) mode, which the initial MS scan analyzes the mass to charge (*m*/*z*) ratios of ions in the mass range 350–1500 Da. The 20 most abundant ions were automatically selected for collision-activated dissociation. All of the MS/MS data were searched against the UniProtKB/Swiss-Prot database using the Mascot program (Matrix Science Ltd. London, UK, version 2.4). 

### 3.19. Statistical Analysis 

The experiments were conducted at least three times, and all of the data are presented as mean ± standard deviation (SD). Statistical analyses were done using the Statistical Package for the Social Sciences (SPSS). The statistical analyses were one-way analysis of variance (ANOVA), followed by Duncan’s Multiple Range tests. A difference was considered statistically significant when *p* < 0.05. 

## 4. Conclusions

In the present study, we found that an extrusion-pretreatment process increased the extraction yield of gelatin from tilapia scale samples. Moreover, TSG2 (preconditioning with 1.26% citric acid) possessed the highest extraction yield, high gelatin purity, a relatively high whiteness index, high bloom gel strength, high viscosity, high odor acceptability, high thermal stability, and high antioxidant activities, indicating the preconditioning of citric acid before extrusion is beneficial in promoting the quality of gelatin. Among the four gelatin hydrolysates generated from TSG2, TSGH4 (digested by pepsin + pancreatin and MWCO < 3000 Da) had a higher yield and exhibited superior antioxidant activities. TSGH4 were further fractionated by Superdex peptide and 2-step RP-HPLC chromatography, which yielded the subfraction containing the most antioxidants, TSGH4-6-2-b. LC-ESI/MS/MS analysis of TSGH4-6-2-b revealed that two peptide sequences (GYDEY and EPGKSGEQGAPGEAGAP) may have potential as novel antioxidant peptides. Our findings provide a method of reusing fish by-product waste, which might be of value in the fishery industries. Further in-depth exploration of these two novel antioxidant peptides, including in vivo studies, is needed. 

## Figures and Tables

**Table 1:** Pretreatment conditions, extrusion variables, extraction variables, and extraction yields of tilapia scale gelatins for TSG1-6.

Pretreatments	TSG1	TSG2	TSG3	TSG4	TSG5	TSG6
Pretreatment solvent	—	—	—	—	0.1 N NaOH	0.1 N NaOH
**Variables of Extrusion**	**TSG1**	**TSG2**	**TSG3**	**TSG4**	**TSG5**	**TSG6**
Preconditioning solvent	1.26% citric acid	1.26% citric acid	9.37% acetic acid	9.37% acetic acid	1.26% acitric acid	1.26% citric acid
Feed moisture (%)	—	27	—	27	—	27
Feed rate (kg/h)	—	11.4	—	11.4	—	11.4
Barrel temperature (°C)	—	135	—	135	—	135
Screw speed (rpm)	—	360	—	360	—	360
Die diameter (mm)	—	3	—	3	—	3
**Variables of Water Extraction**	**TSG1**	**TSG2**	**TSG3**	**TSG4**	**TSG5**	**TSG6**
Extraction temperature (°C)	50	50	50	50	50	50
Extraction time (h)	1	1	1	1	1	1
**Extraction Yield of Gelatins**	**TSG1** ^**2**^	**TSG2** ^**2**^	**TSG3** ^**2**^	**TSG4** ^**2**^	**TSG5** ^**2**^	**TSG6** ^**2**^
Extraction yield (%) ^1^	2.10 ± 0.03 ^a^	22.2 ± 1.0 ^d^	2.18 ± 0.09 ^a^	19.3 ± 0.4 ^c^	2.13 ± 0.03 ^a^	13.0 ± 0.2 ^b^

—, not adopted; ^1^ The values are expressed as g protein/100 g crude protein content of tilapia fish scales, dry basis; ^2^ Values are mean ± SD (n = 3); values in the same row with varying letters (^a^, ^b^, ^c^, and ^d^) differ (*p* < 0.05).

**Table 2:** Physicochemical properties of TSG2, TSG4, and TSG6.

Physicochemical Properties	TSG2 ^1^	TSG4 ^1^	TSG6 ^1^
*L*	82.3 ± 0.0 ^b^	74.5 ± 0.1 ^a^	93.2 ± 0.1 ^c^
*a*	0.63 ± 0.03 ^b^	1.52 ± 0.03 ^c^	−1.39 ± 0.04 ^a^
*b*	9.27 ± 0.09 ^b^	10.0 ± 0.0 ^c^	5.42 ± 0.02 ^a^
Whiteness index	80.0 ± 0.0 ^b^	72.5 ± 0.0 ^a^	91.2 ± 0.0 ^c^
Ash (%)	1.75 ± 0.06 ^a^	3.58 ± 0.27 ^b^	3.33 ± 0.09 ^b^
pH	7.2	6.2	9.9
Bloom gel strength (g)	185 ± 5 ^b^	157 ± 5 ^a^	202 ± 0 ^c^
Viscosity (cps)	9.77 ± 0.12 ^b^	8.10±0.24 ^a^	9.53 ± 0.17 ^b^
*T*_max1_ (°C)	79.0	57.9	62.6
*T*_max2_ (°C)	176	182	179
H1 (J/g)	1.40	8.01	3.37
H2 (J/g)	116	166	152
Odor (in solution) ^2^	5.21 ± 1.68 ^b^	4.04 ± 2.07 ^a^	5.38 ± 1.60 ^b^
Odor (in powder) ^2^	6.08 ± 1.15 ^b^	3.17 ± 1.97 ^a^	6.46 ± 1.08 ^b^
Overall acceptability ^2^	6.58 ± 1.55 ^b^	5.08 ± 1.85 ^a^	5.42 ± 1.35 ^a^

^1^ Values are mean ± SD (n = 3); values in the same row with varying letters (in ^a^, ^b^, and ^c^) differ (*p* < 0.05). ^2^ Sensory attributes were rated on a nine-point hedonic scoring scale (1 = very bad, 5 = moderate, 9 = very good).

**Table 3:** Variables involved in the preparation of gelatin hydrolysates, molecular weight distribution, and antioxidant activities of TSGH1, TSGH2, TSGH3, and TSGH4.

Process Variables	TSGH1	TSGH2	TSGH3	TSGH4
Gelatin source	TSG2	TSG2	TSG2	TSG2
Enzyme used	Pepsin	Pepsin + Pancreatin	Pepsin	Pepsin + Pancreatin
Digestion conditions	pH 2.0, 37 °C, 4 h	pH 2.0, 37 °C, 4 h (for Pepsin); pH 7.0, 37 °C, 4 h (for Pancreatin)	pH 2.0, 37 °C, 4 h	pH 2.0, 37 °C, 4 h (for Pepsin); pH 7.0, 37 °C, 4 h (for Pancreatin)
Ultrafiltration condition	>3000 Da	>3000 Da	<3000 Da	<3000 Da
**Molecular Weight (MW)**	**TSGH1**	**TSGH2**	**TSGH3**	**TSGH4**
Peak MW ^1^ (Da)	24,012	18,006	131	1315
MW interval (Da)	2883–37,466	1800–37,466	96–1184	74–21,625
**Antioxidant Activities** ^**1**^	**TSGH1** ^**2**^	**TSGH2**	**TSGH3**	**TSGH4**
DPPH scavenging activity (%)	43.3 ± 1.1 ^a 3^	44.4 ± 3.6 ^a^	84.0 ± 0.3 ^c^	71.8 ± 2.5 ^b^
TEAC (μM)	12.7 ± 0.1 ^a^	29.7 ± 0.4 ^c^	27.6 ± 0.2 ^b^	29.2 ± 0.3 ^c^
Absorbance (700 nm)	0.15 ± 0.02 ^a^	0.24 ± 0.01 ^b^	0.20 ± 0.05 ^a^	0.23 ± 0.01 ^b^

^1^ Peak MW: molecular weight of the highest peak; ^2^ The concentration for TSGH1-4 was 10 mg/mL. ^3^ Values are mean ± SD (n = 3); values in the same row with different letters (in ^a^, ^b^, and ^c^) differ significantly (*p* < 0.05).

**Table 4:** Peptide content and DPPH scavenging activity of fractionated products from TSGH4.

Samples	Peptide Content (μg/μL) ^1^	DPPH Scavenging Activity (Vit C ppm/μg Peptide) ^1^
TSGH4 separated by Superdex peptide column		
TSGH4-1	0.13 ± 0.00	0.12 ± 0.03
TSGH4-2	0.08 ± 0.00	0.00 ± 0.00
TSGH4-3	0.08 ± 0.00	0.00 ± 0.00
TSGH4-4	0.15 ± 0.00	0.11 ± 0.06
TSGH4-5	0.13 ± 0.00	0.43 ± 0.06
TSGH4-6	0.16 ± 0.05	0.97 ± 0.03
TSGH4-7	0.31 ± 0.00	0.23 ± 0.02
TSGH4-8	0.15 ± 0.00	0.53 ± 0.03
TSGH4-9	0.13 ± 0.00	0.00 ± 0.00
TSGH4-10	0.69 ± 0.01	0.35 ± 0.01
TSGH4-11	0.15 ± 0.00	0.48 ± 0.02
TSGH4-12	0.14 ± 0.00	0.00 ± 0.00
TSGH4-6 separated by RP-HPLC C18 column		
TSGH4-6-1	0.097 ± 0.003	1.17 ± 0.05
TSGH4-6-2	0.085 ± 0.002	1.17 ± 0.06
TSGH4-6-1 separated by RP-HPLC C18 column		
TSGH4-6-1-a	0.086 ± 0.003	1.22 ± 0.14
TSGH4-6-1-b	0.089 ± 0.004	1.35 ± 0.15
TSGH4-6-1-c	0.082 ± 0.003	0.80 ± 0.08
TSGH4-6-2 separated by RP-HPLC C18 column		
TSGH4-6-2-a	0.086 ± 0.004	1.13 ± 0.19
TSGH4-6-2-b	0.090 ± 0.004	1.60 ± 0.22
TSGH4-6-2-c	0.092 ± 0.003	1.39 ± 0.09

^1^ Values are mean ± SD (n = 3).

**Table 5:** A selection of the most intense ions corresponding to peptides in TSGH4-6-2-b by LC-ESI/MS/MS.

Protein Origin	Molecular Mass (+1)	Peptide Sequence
Collagen alpha-2(I) chain	440.24	GPLGP
	442.26	GPVGL
	470.25	LPGSP
	511.28	GPAGPL
	543.27	GELGPA
	568.30	GPAGPLG
	592.27	GPSGFAG
	646.23	GYDEY
	778.40	GLPGPPGPS
	1194.57	ADGNTGPAGPAGPL
	1251.59	GADGNTGPAGPAGPL
	1268.59	GPAGARGADGNTGPA
	1349.60	GSPGPDGNNGPAGPVG
Collagen type I alpha 1	440.24	GPIGP
	470.25	LPGPS
	511.28	GPAGIP
	543.27	GEIGPA
	568.30	QPGLPG
	568.30	GPAGIPG
	610.31	VGPPGPS
	994.50	GPAGASGPAGPR
	1075.51	GAPGPPGPSGPQG
	1307.62	GETGPAGVPGPAGPSG
	1330.67	PGPAGATGAPGPQGPV
	1538.70	EPGKSGEQGAPGEAGAP

## Data Availability

Data are contained within the article.

## Supplementary Materials

The following are available online at https://www.mdpi.com/article/10.3390/md19050275/s1, Figure S1: Size exclusion chromatographic profiles for TSGH1, TSGH2, TSGH3, and TSGH4. Trypsin, aprotinin, glutathione, Gly-Gly-Gly, and glycine were utilized as the standard proteins.
