# Supplementary material for: Physicochemical and Antioxidant Properties of Gelatin and Gelatin Hydrolysates Obtained from Extrusion-Pretreated Fish (Oreochromis sp.) Scales
Source: Mar Drugs. 2021 May 14;19(5):275. doi: 10.3390/md19050275 (PMC8156103; doi:10.3390/md19050275)

Supplementary Materials

Figure S1. Size exclusion chromatographic profiles for TSGH1, TSGH2, TSGH3, and TSGH4. Trypsin, aprotinin, glutathione, Gly-Gly-Gly, and glycine were utilized as the standard proteins.

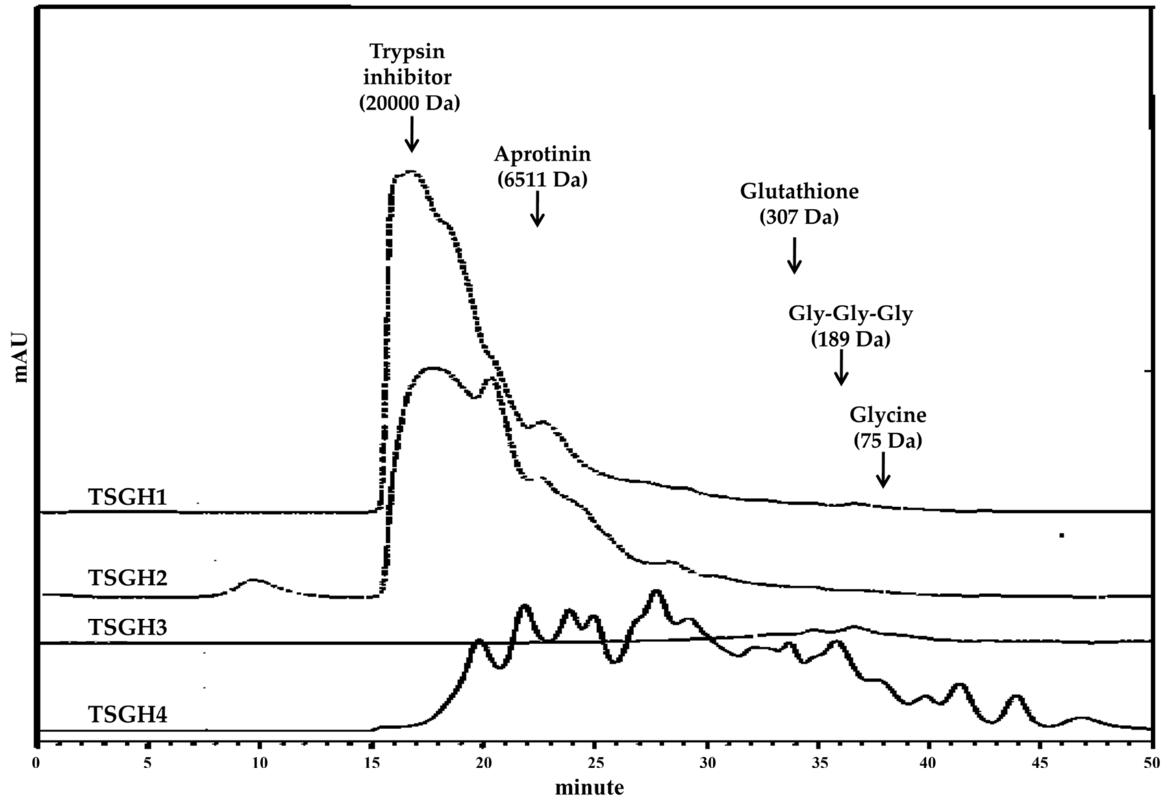

Supplement: Supplementary file 1 [file marinedrugs-19-00275-s001.zip › marinedrugs-1212504-supplementary.pdf]
